# Molecular determinants of 2-aminoethoxydiphenyl borate sensitivity of transient receptor potential vanilloid 2-unexpected differences between 2 rodent orthologs

**DOI:** 10.1016/j.molpha.2025.100060

**Published:** 2025-07-11

**Authors:** Tabea C. Fricke, Anna Rämisch, Ruth A. Pumroy, Sebastian Pantke, Christine Herzog, Frank G. Echtermeyer, Samer Al-Samir, Volker Endeward, Vera Moiseenkova-Bell, Andreas Leffler

**Affiliations:** 1 Department of Anesthesiology and Intensive Care Medicine, Hannover Medical School, Hannover, Germany; 2 PRACTIS Clinician Scientist Program, Dean’s Office for Academic Career Development, Hannover Medical School, Hannover, Germany; 3 Department of Systems Pharmacology and Translational Therapeutics, Perelman School of Medicine, University of Pennsylvania, Philadelphia; 4 Department of Molecular and Cellphysiology, AG Vegetative Physiologie, Hannover Medical School, Hannover, Germany

**Keywords:** Transient receptor potential vanilloid 2, Species differences, 2-aminoethoxydiphenyl borate, Binding site

## Abstract

Transient receptor potential vanilloid 2 (TRPV2) is relevant for diseases like cancer, cardiac dysfunction, and infection, warranting drug development targeting TRPV2. However, this has been complicated by the lack of good modulators targeting TRPV2 and questions about species selectivity, so more detailed molecular insights into channel function and pharmacology are required. Two recent studies identified distinct binding sites on rat (r) and mouse (m) TRPV2 for activation by 2-aminoethoxydiphenyl borate (2-APB). Here we aimed to determine whether the mechanisms for 2-APB sensitivity of TRPV2 indeed differ among these closely related orthologs. Patch clamp electrophysiology revealed that mTRPV2 and human TRPV2 display similar sensitivities to 2-APB when compared with a considerably higher sensitivity of rTRPV2. For both mTRPV2 and rTRPV2, we observed that the exchange of putative 2-APB binding residues within the vanilloid binding pocket alters overall channel sensitivity to 3 TRPV2 pharmacological activators that bind at different sites: 2-APB, cannabidiol, and probenecid. By contrast, the exchange of putative 2-APB binding residues at the S5 binding pocket in both channels resulted in strongly reduced 2-APB sensitivies without reducing sensitivity to cannabidiol and probenecid. rTRPV2 mutants lacking key residues of both binding sites were almost completely 2-APB insensitive. These functional data suggest that the mechanisms accounting for 2-APB sensitivity are similar across mammalian TRPV2 orthologs. Except for serving as a binding site for 2-APB, the vanilloid binding pocket plays a key role in the overall function of TRPV2. These findings are relevant for the emerging framework toward an improved understanding of TRPV2.

**Significance Statement:**

This study resolves the conflict regarding how 2-aminoethoxydiphenyl borate binds to transient receptor potential vanilloid 2 (TRPV2), showing a shared mechanism despite sensitivity differences. These findings enhance TRPV2 modulation insights and highlight species considerations in drug design, aiding the development of selective TRPV2-targeted therapies.

## Graphical abstract

**Figure:**
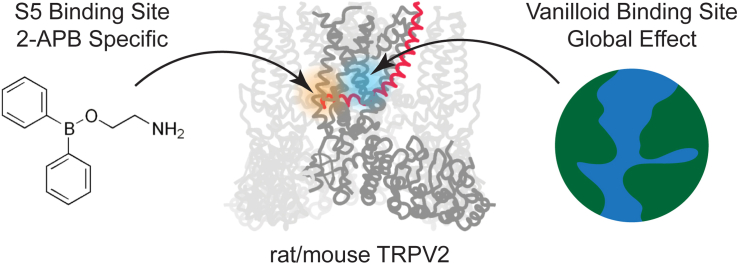

## 1 Introduction

Transient receptor potential vanilloid 2 (TRPV2) is a Ca^2+^-permeable, nonselective cation channel that is part of the vanilloid subfamily within the transient receptor potential channel family.^1^ TRPV2 is still rather enigmatic, but recent studies have suggested its crucial role in several physiological functions including myocardial structure and function, innate immunity, neuronal outgrowth, sensory neuron mechanosensitivity, endocrine secretion, endometrial development, and thermogenesis in brown fat.^2^, ^3^, ^4^, ^5^, ^6^, ^7^, ^8^, ^9^, ^10^, ^11^ In terms of pathophysiology, TRPV2 seems to be important for the growth and invasiveness of various tumors, for the development of cardiomyopathy, and in the context of bacterial or virus infections.^12^, ^13^, ^14^, ^15^, ^16^, ^17^, ^18^

One major challenge when studying TRPV2 is the lack of specific agonists. Compounds such as 2-aminoethoxydiphenyl borate (2-APB) and cannabidiol (CBD) are commonly used to activate TRPV2, but these substances are nonspecific and affect many other proteins, including the closely related TRPV1 and TRPV3.^19^, ^20^, ^21^, ^22^ Nevertheless, they are important model substances allowing mechanistic and pharmacological studies on TRPV2. Recent reports have combined structural and functional techniques to identify the molecular determinants for sensitization and activation of rat (r), mouse (m), or rabbit TRPV2 by endogenous and exogenous ligands including 2-APB and CBD, but also the cannabinoid C16, the uricosuric drug probenecid, the vanilloid resiniferatoxin, weak acids, and cholesterol.^1^^,^^22^, ^23^, ^24^, ^25^, ^26^, ^27^, ^28^, ^29^, ^30^

Despite being commonly used to examine TRPV2 function, the binding site and mechanism for 2-APB have proved controversial. Three groups, including our own, have published cryo-election microscopy structures of TRPV2 in the presence of 2-APB with 3 different results. First, our group suggested binding of 2-APB in a pocket between the S5 helix and the S4-S5 linker of 2 adjacent monomers (S5 binding pocket).^22^ The mutations of residues His521 or Arg539 within this pocket resulted in a prominent rightward shift of the concentration-response relationship, but even the double-mutant rTRPV2-His521Ala/Arg539Lys still produced large inward currents when exposed to high concentrations of 2-APB.^22^ We have observed the same 2-APB binding site in 2 subsequent papers at higher resolution.^29^^,^^30^ Next, Su et al^27^ suggested a competitive binding of 2-APB and cholesterol within the vanilloid binding pocket (VBP) of mTRPV2. The authors demonstrated that some mTRPV2-mutant constructs with single replacements of predicted binding residues within the VBP (mTRPV2-Tyr466Ala, -Gln525Asp, -Leu627Ala) were completely insensitive to 2-APB, though alternate substitutions at the key residue Gln525 remained sensitive to 2-APB (mTRPV2-Gln525Ala, -Gln525Phe, -Gln525Thr).^27^ Finally, Gochman et al^31^ were unable to observe density for 2-APB in their structures of rTRPV2 with the channel exposed to both CBD and 2-APB.

**Fig. 1:**
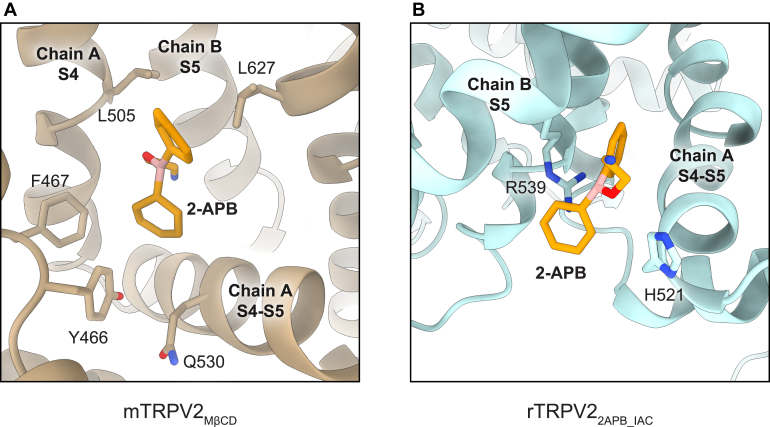
(A) 2-APB binding site in mTRPV2 (pdb 7YEP). Residues proposed to interact with 2-APB are shown in addition to residues mutated to imbue RTX sensitivity. Residue numbering is for mTRPV2. (B) 2-APB binding site in rTRPV2 (pdb 7N0N). Residues proposed to interact with 2-APB are shown with residue numbers for rTRPV2.

Due to the inconsistency between the 2 identified binding sites, Su et al^27^ suggested a not-yet-appreciated species-specific difference between rTRPV2 and mTRPV2 (Fig. 1). While reviewing the structures supporting these sites, we noticed that the proposed densities for 2-APB in the mTRPV2 structures were more elongated than expected for 2-APB, but instead similar to densities commonly assigned to lipids. The 2-APB molecule has a lobed triangular shape which has been observed consistently in density assigned to 2-APB in other published structures across a similar resolution range (Supplemental Fig. 1). Based on this observation, it is unclear whether the reported discrepancy between sites reflects a real difference between species or is the result of a misidentified drug-binding pocket.

This study aims to comprehensively examine the role of the 2 predicted 2-APB binding sites to gain a better understanding of possible species specificity. We rigorously re-examined 2-APB-induced activation of both rTRPV2 and mTRPV2 by employing patch clamp electrophysiology to investigate multiple mutant constructs.

## 2 Materials and methods

### 2.1 Chemicals

All chemicals were diluted immediately prior to use. 2-APB was obtained from Tocris (Bio-Technology) and stored at –8 °C in a 100 mM stock in DMSO. CBD and methyl-*β*-cyclodextrin (M*β*CD) was purchased from Cayman Chemical Co. CBD was kept at 8 °C and MβCD were stored in DMSO at –20 °C. Probenecid from AAT Bioquest was dissolved in external solution and stored at –8 °C.

### 2.2 Cell culture

HEK 293T cells were transfected with various plasmids using jetPEI (Polyplus-transfection SA). The cDNA of mTRPV2 was kindly donated by Dr Itaru Kojima (Gunma University) and the cDNA of rTRPV2 was generously provided by Dr Michael Caterina (Johns Hopkins University School of Medicine). Cells were cultured under standard conditions (5% CO_2_ at 37 °C) in Dulbecco’s modified Eagle medium/nutrient mixture F12 (Gibco/Invitrogen) supplemented with 10% FBS (Biochrom).

Mutants of mTRPV2 (Q525N, Q525A, Y466A, L627A, and H516A) and rTRPV2 (Q530A, Q530N, Y471A, L632A, H521A, H521A/R529K, H521A/Q530N/R539K, H521A/R539K/L632A, H521A/Q530N/R539K/L632A, F472S/L510T, and F472S/L510T/Q530E) (see the sequence alignment in Supplemental Fig. 2) were generated via site-directed mutagenesis using the QuikChange Lightning Site-Directed Mutagenesis Kit (Agilent) following the manufacturer’s instructions. All mutants were sequenced to confirm the intended amino acid substitutions and to ensure no additional mutations were present.

Stably TRPV2-expressing HEK 293 cells were created by transfection of the vector encoding for rTRPV2, mTRPV2, or human (h) TRPV2 with a selection antibiotic tag with jetPEI following the manufacturer’s instructions. Twenty-hours posttransfection cells were cultured in Dulbecco’s modified Eagle medium F12 with 10% FBS containing the selection antibiotic to select for stable integrands. Individual colonies were transferred to a 24-well plate and expanded under continuous antibiotic selection. To verify functional expression calcium imaging experiments and patch clamp recordings were performed to assess TRPV2 activity in response to 2-APB. Approximately 24 hours posttransfection, cells were washed using phosphate-buffered saline (Lonza), detached with trypsin/EDTA in phosphate-buffered saline (0.05%, Bio & Sell), and seeded for patch clamp experiments.

### 2.3 Cholesterol depletion and quantification

To confirm the effective depletion of membrane cholesterol, HEK293t cells were treated with 10 mM M*β*CD for 15 minutes at 37 °C in external solution. Following treatment, cells were washed twice with external solution and harvested.

Membrane cholesterol levels were quantified using the Amplex Red Cholesterol Assay Kit (Invitrogen, Thermo Fisher Scientific) following the manufacturer’s instructions. Fluorescence intensities were measured using a FLUOstar Optima microplate reader (BMG Labtech). To normalize lipid levels across samples, total protein concentrations were measured using the Roti-Nanoquant protein assay (Carl Roth GmbH), and lipid concentrations were expressed relative to total protein content. Comparisons were made between M*β*CD-treated and untreated cells to evaluate the extent of cholesterol sequestration.

### 2.4 Patch clamp

Whole-cell voltage-clamp experiments were conducted on cells expressing mTRPV2, rTRPV2, and hTRPV2. Signals were low-pass filtered at 1 kHz and sampled at 2–10 kHz using an EPC10 USB HEKA amplifier (HEKA Elektronik) during these experiments. Patch pipettes had a resistance of 2.0–5.0 MΩ and were made from borosilicate glass tubes (TW150F-3; World Precision Instruments). Only cells with an initial series resistance <10 MOhm were used for experiments. No series resistance compensation was performed. The standard external solution contained (in mM) 140 NaCl, 5 KCl, 2 MgCl_2_, 5 EGTA, 10 HEPES, and 10 glucose (pH adjusted to 7.4 with NaOH). The pipette solution contained (in mM) 140 KCl, 2 MgCl_2_, 5 EGTA, and 10 HEPES (pH adjusted to 7.4 with KOH). All recordings were performed at room temperature and cells were held at –60 mV. Solutions were bath applied using a gravity-driven polytetrafluoroethylene/glass multibarrel perfusion system. Data acquisition and offline analyses were performed using Patchmaster software (HEKA Elektronik) and Origin 8.5.1 (Origin Lab, Northampton). For all dose-response analyses, current amplitudes were normalized to the maximum current observed in each individual recording, regardless of drug concentration. As a result, the normalized response at the highest tested concentration may occasionally appear below 1.0 if a lower concentration elicited the maximal current in that particular cell. This normalization approach was chosen to account for variability in absolute current densities across cells and recordings.

### 2.5 Statistical analysis

All data are presented as the mean ± SEM plus scatter plots. Box-and-whisker plots were used to visualize data distributions. Each box represents the interquartile range, spanning from the 25th to the 75th percentile, with the median (50th percentile) shown as a horizontal line. Whiskers extend to the 5th and 95th percentiles. Data points outside of this range are plotted individually. This definition of whiskers differs from Tukey’s 1.5× interquartile range method and was chosen to better represent the full range of typical values in the dataset. Statistical analysis was performed using GraphPad Prism 9 (GraphPad). Comparison between groups was determined using a 2-tailed 2-sample t-test or ANOVA with a post hoc Tukey test. The details of the tests and sample sizes for each experiment are provided in the figure legends. In this manuscript, we use the term “statistically significant' exclusively to indicate results with a *P*-value <.05, unless otherwise specified. Where relevant, we also report effect sizes and 95% confidence intervals (CIs) to provide context on the magnitude and precision of the observed differences.

## 3 Results

### 3.1 Effect of cholesterol on TRPV2 orthologs

**Fig. 2:**
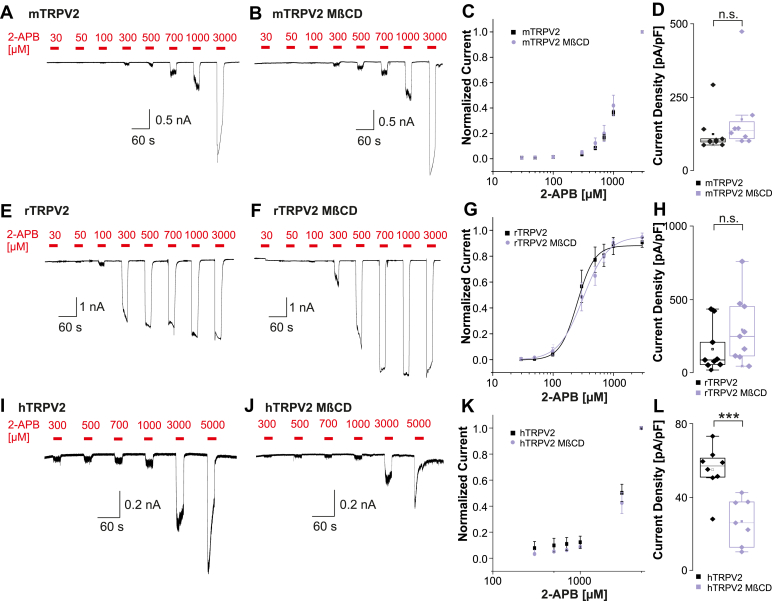
Cholesterol depletion does not increase 2-APB sensitivity of TRPV2. (A, B, E, F, I, J) Patch clamp traces showing 2-APB-induced concentration-dependent activation of mTRPV2 (A, B), mTRPV2 (E, F), and hTRPV2 (I, J) without or with pretreatment with M*β*CD. (C, G, and K) Concentration-response curves for responses induced by increasing concentrations of 2-APB. Current amplitudes were measured at each concentration and normalized to the maximum amplitude. Data are presented as mean ± SEM, and the drawn lines represent fits with the Hill equation. (D, H, and L) Box plots displaying current densities of 2-APB-induced currents on mTRPV2 (D), rTRPV2 (H), and hTRPV2 (L). Boxes represent the median (50th percentile) along with the 25th and 75th percentiles, whereas the whiskers indicate the 5th and 95th percentiles. Data points outside of the whiskers are plotted individually. ∗*P* < .05; ∗∗*P* < .01; ∗∗∗*P* < .001; and n.s. not significant.

Su et al^27^ suggested that cholesterol bound within the VBP inhibits mTRPV2 and that 2-APB activates by displacing cholesterol and then binding to the VBP. As we and others have not observed cholesterol binding to the rat VBP,^22^ we examined the effect of depletion of membrane cholesterol on commonly studied TRPV2 orthologs.^21^^,^^22^^,^^24^^,^^29^^,^^31^^,^^32^ HEK 293 cells stably expressing mTRPV2, rTRPV2, or hTRPV2 were investigated following depletion of cholesterol with 10 mM M*β*CD for 15 minutes.^27^^,^^33^^,^^34^ This treatment resulted in an average reduction of cellular cholesterol content by approximately 27% (*n* = 3, Student’s *t* test, *P* = .008, 95% CI: –42.41 to –12.05, Supplemental Fig. 3A). Higher concentrations of M*β*CD were not used, as they compromised cell integrity and prevented successful patch-clamp recordings. In contrast to the results of Su et al,^27^ we did not observe a substantial shift of the sensitivity of mTRPV2 to 2-APB in M*β*CD-pretreated cells as compared with untreated cells (*n* = 8–9, Fig. 2, A–C). Also, the maximal currents amplitudes induced by 2-APB were not significantly higher in the M*β*CD-pretreated cells (Student’s *t* test, *P* = .3, 95% CI: –50.4 to 151.3, Fig. 2D). The concentration-response curve for rTRPV2 even showed a slight rightward shift following M*β*CD (EC_50_ rTRPV2 = 254 ± 12.9 *μ*M, *n* = 9; EC_50_ rTRPV2_MβCD_ = 316.3 ± 19.3 *μ*M, *n* = 9, Fig. 2, E–G). The mean current density of 2-APB-induced currents appeared larger in M*β*CD-treated cells, but this difference did not reach statistical significance (Student’s *t* test, *P* = .173, 95% CI: –64.49 to 329.7, Fig. 2H). Although hTRPV2 has been reported to be largely 2-APB insensitive,^25^ we found that increasing concentrations of 2-APB from 300 to 5000 *μ*M induced small but reversible inward currents in untreated as well as in M*β*CD-treated cells, but not in naïve HEK293 cells (Fig. 2, I and J, *n* = 7–8, Supplemental Fig. 3B). The small currents not reaching saturation at 5000 *μ*M 2-APB hindered us from determining valid EC_50_ values, but the resulting concentration-response plot did not reveal any substantial differences (Fig. 2K). In contrast to mTRPV2 and rTRPV2, the mean current density of currents for hTRPV2 induced by 2-APB was significantly reduced in cells depleted from cholesterol (Fig. 2L, Student’s *t* test, *P* = .001, 95% CI: –42.6 to –13.78). Taken together, these data do not show the expected increase of 2-APB sensitivity in any of the 3 TRPV2 orthologs following depletion of cholesterol. As a consequence, our data do not support the notion that cholesterol might regulate 2-APB sensitivity due to competitive binding to the VBP. However, the data demonstrate that hTRPV2 is not 2-APB insensitive, but displays a 2-APB sensitivity that is comparable to that of mTRPV2.

### 3.2 Modification of 2-APB binding residues within the VBP

**Fig. 3:**
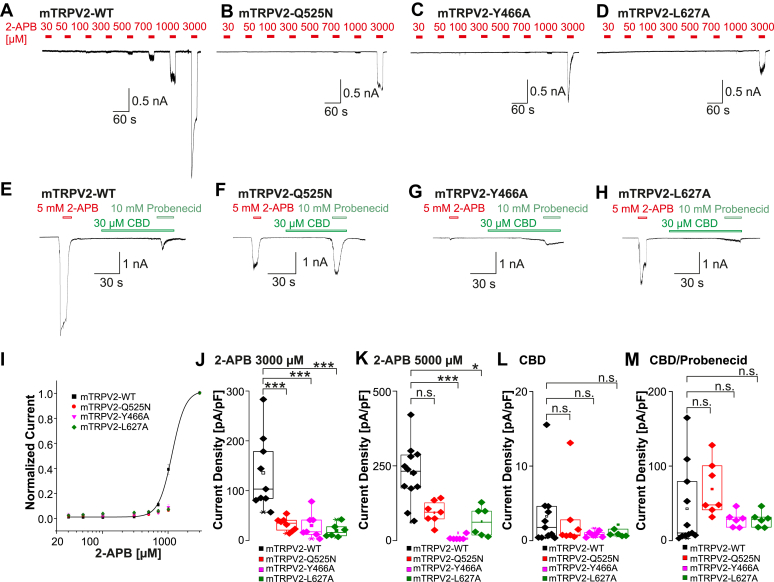
Modification of 2-APB binding residues within the mTRPV2 VBP results in decreased sensitivity of 2-APB, CBD, and probenecid. (A–D) Patch clamp traces showing concentration-dependent activation of mTRPV2-WT (A), -Q525N (B), -Y466A (C), and L627A (D) by 2-APB. Increasing concentrations of 2-APB were applied and cells were held at –60 mV. (E–H) Samples of patch clamp recordings displaying activation by 5 mM 2-APB followed by 30 *μ*M CBD and the coapplication with 10 mM probenecid in mTRPV2-WT (E) -Q525N (F), -Y466A (G), -L627A (H). (I) Concentration-response curves for experiments displayed in panels A through D. Current amplitudes were measured at each concentration and normalized to the maximum amplitude. (J–M) Comparisons of current densities evoked by 3000 *μ*M 2-APB (J), 5000 *μ*M 2-APB (K), 30 *μ*M CBD (L), or 30 *μ*M CBD + 10 mM probenecid (M). Boxes represent the median (50th percentile) along with the 25th and 75th percentiles, whereas the whiskers indicate the 5th and 95th percentiles. Data points outside of the whiskers are plotted individually. ∗*P* < .05; ∗∗*P* < .01; ∗∗∗*P* < .001; and n.s. not significant. *P*-values from the post hoc Tukey test are shown on the plots.

We next examined if the 2-APB sensitivities of mTRPV2 and rTRPV2 are dictated by the predicted residues within the VBP (Supplemental Fig. 2). First, we performed experiments on mTRPV2 mutants that were previously reported to exhibit a completely abolished response to 2 mM 2-APB.^27^ Instead of only examining one concentration of 2-APB, cells held at –60 mV were treated with increasing concentrations of 2-APB (30–3000 *μ*M) to create concentration-response curves for mTRPV2-wild type (WT) (*n* = 9), mTRPV2-Q525N (*n* = 8), -Y466Ala (*n* = 7), or -Leu627Ala (*n* = 6). All 3 mutants only produced substantial inward currents at 3000 *μ*M 2-APB, making it impossible to calculate EC_50_ values (Fig. 3, A–D and I). These currents were significantly smaller than those observed for mTRPV2-WT (Fig. 3, A and J, one-way ANOVA, F(3,26) = 12, Tukey Honestly Significant Difference (HSD) post hoc test, WT vs Q525N 95% CI: 44.73 to 161.6; WT vs Y466A 95% CI: 45.17 to 166.3; WT vs L627A 95% CI: 52.52 to 179.2). These data confirm that all 3 mutants indeed exhibit strongly reduced 2-APB sensitivities. However, they are not completely 2-APB insensitive as was previously suggested.^27^

We extended the characterization of these mutants by assessing their maximal responses to the commonly used TRPV2 agonists—5000 *μ*M 2-APB, 30 *μ*M CBD, and 10 mM probenecid—to evaluate whether the mutants only affect 2-APB sensitivity or rather overall channel function. Both CBD and probenecid employ binding sites on TRPV2 distinct from those proposed for 2-APB, and their combined application strongly activates rTRPV2.^30^ These supramaximal concentrations did not induce currents in naïve HEK 293t cells (Supplemental Fig. 3B). Although significantly reduced when compared with large inward currents produced by mTRPV2-WT (Fig. 3E, *n* = 12), the mutants mTRPV2-Q525N (Fig. 3F, *n* = 7) and mTRPV2-L627A (Fig. 3H, *n* = 6) produced large inward currents when challenged with 5000 *μ*M 2-APB (Fig. 3K, one-way ANOVA F(3,27) = 8.5, Tukey HSD post hoc test, WT vs Q525N 95% CI: –19.04 to 132.3; WT vs Y466A 95% CI: 61.86 to 220.9; WT vs L627A 95% CI: 6.792 to 165.8). Only mTRPV2-Y466A almost completely failed to respond to 5000 *μ*M 2-APB (Fig. 3G, *n* = 6). To our surprise, most of the investigated cells with mTRPV2-WT produced small or no currents when exposed to 30 *μ*M CBD alone or in combination with 10 mM probenecid (Fig. 3, E, L, and M, *n* = 11). CBD-induced currents were not significantly smaller for any of the 3 mutants (Fig. 3L, *n* = 6–11, one-way ANOVA, F(3,26) = 0.5, Tukey HSD post hoc test, WT vs Q525N 95% CI: –4.6 to 5.383; WT vs Y466A 95% CI: –2.87 to 7.608; WT vs L627A 95% CI: –4.126 to 6.353), and only mTRPV2-Q525N produced robust inward currents induced by the combination of CBD and probenecid (Fig. 3M, *n* = 6–11, one-way ANOVA F(3,26) = 3.3, Tukey HSD post hoc test, WT vs Q525N 95% CI: –76.66 to 23.82; WT vs Y466A 95% CI: –38.39 to 67.07; WT vs L627A 95% CI: –14.3 to 91.17). These data suggest that all 3 mutants are to some degree functional. However, at least mTRPV2-Y466A and mTRPV2-L627A seem to have generally reduced responses. These mutant phenotypes prevent us from concluding that these residues are specifically important for 2-APB sensitivity.

**Fig. 4:**
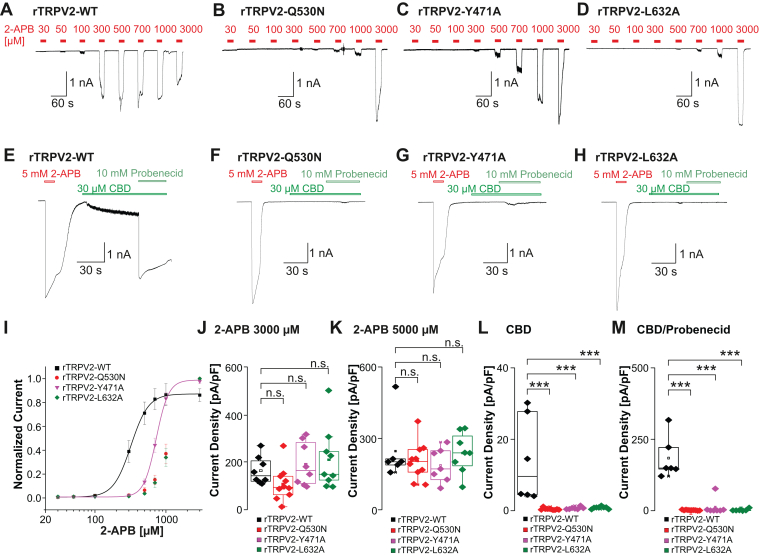
Modification of 2-APB binding residues within the rTRPV2 VBP results in decreased sensitivity of 2-APB, CBD, and probenecid. (A–D) Patch clamp traces showing concentration-dependent activation of rTRPV2-WT (A), -Q530N (B), -Y471A (C), and L632A (D) by 2-APB. Increasing concentrations of 2-APB were applied and cells were held at –60 mV. (E–H) Samples of patch clamp recordings displaying activation by 5 mM 2-APB followed by 30 *μ*M CBD and the coapplication with 10 mM probenecid in rTRPV2-WT(E), -Q530N (F), -Y471A (G), -L632A (H). (I) Concentration-response curves for experiments displayed in panels A through D. Current amplitudes were measured at each concentration and normalized to the maximum amplitude. (J–M) Comparisons of current densities evoked by 3000 *μ*M 2-APB (J), 5000 *μ*M 2-APB (K), 30 *μ*M CBD (L), or 30 *μ*M CBD + 10 mM probenecid (M). Boxes represent the median (50th percentile) along with the 25th and 75th percentiles, whereas the whiskers indicate the 5th and 95th percentiles. Data points outside of the whiskers are plotted individually. ∗*P* < .05; ∗∗*P* < .01; ∗∗∗*P* < .001; and n.s. not significant. *P*-values from the post hoc Tukey test are shown on the plots.

We next generated corresponding mutants on rTRPV2 (-Q530N, -Y471A, and -L632A, Supplemental Fig. 2) and created concentration-response curves for 2-APB (Fig. 4, A–D and I). All 3 mutants (-Q530N, *n* = 10; -L632A, *n* = 9; -Y417A, *n* = 8) generated robust inward currents at 2-APB concentrations >700 *μ*M, but the corresponding concentration-response curves showed a pronounced rightward shift when compared with rTRPV2-WT (EC_50_ : 322 ± 4 *μ*M, *n* = 8, Fig. 4I). As these curves did not clearly reach saturation within the tested concentration range, we did not determine EC_50_ values for the mutants. Surprisingly, the mean current densities of the largest responses to 2-APB did not differ between WT and mutant channels (Fig. 4J, *n* = 8–10, one-way ANOVA F(3,31) = 0.4, Tukey HSD post hoc test, WT vs Q530N 95% CI: –127.1 to 127.5; WT vs Y417A 95% CI: –161.2 to 107.2; WT vs L632A 95% CI: –176.3 to 84.46). The same result was observed with a single application of 5000 *μ*M 2-APB, eg, all 3 mutants produced large inward currents that were not significantly different from currents produced by rTRPV2-WT (Fig. 4, E–H and K, *n* = 6–10, one-way ANOVA, F(3,27) = 0.6, Tukey HSD post hoc test, WT vs Q530N 95% CI: –87.3 to 170.7; WT vs Y417A 95% CI: –79.43 to 198.5; WT vs L632A 95% CI: –126.7 to 143.1). Although large inward currents were induced by 30 *μ*M CBD in cells with rTRPV2-WT (Fig. 4, E and L), all 3 mutants displayed diminished CBD sensitivities (Fig. 4, F–H and L, *n* = 6–10, one-way ANOVA F(3,27) = 11, Tukey HSD post hoc test, WT vs Q530N 95% CI: 6.471 to 21.17; WT vs Y417A 95% CI: 5.704 to 21.54; WT vs L632A 95% CI: 5.775 to 21.14). The combination of CBD and probenecid induced large inward currents on rTRPV2-WT, but again all 3 mutants almost completely failed to produce currents when exposed to CBD + probenecid (Fig. 4, E–H and M, one-way ANOVA, F(3,25) = 39.5, Tukey HSD post hoc test, WT vs Q530N 95% CI: 131.1 to 233.3; WT vs Y417A 95% CI: 117.1 to 227.1; WT vs L632A 95% CI: 124.8 to 239). These data show that an exchange of the investigated residues within the VBP results in a general effect on rTRPV2 channel function as well, and not in a specific reduction of 2-APB sensitivity. These data also reveal rather unexpected differences between mTRPV2 and rTRPV2 regarding their sensitivity to CBD (rTRPV2 >> mTRPV2).

**Fig. 5:**
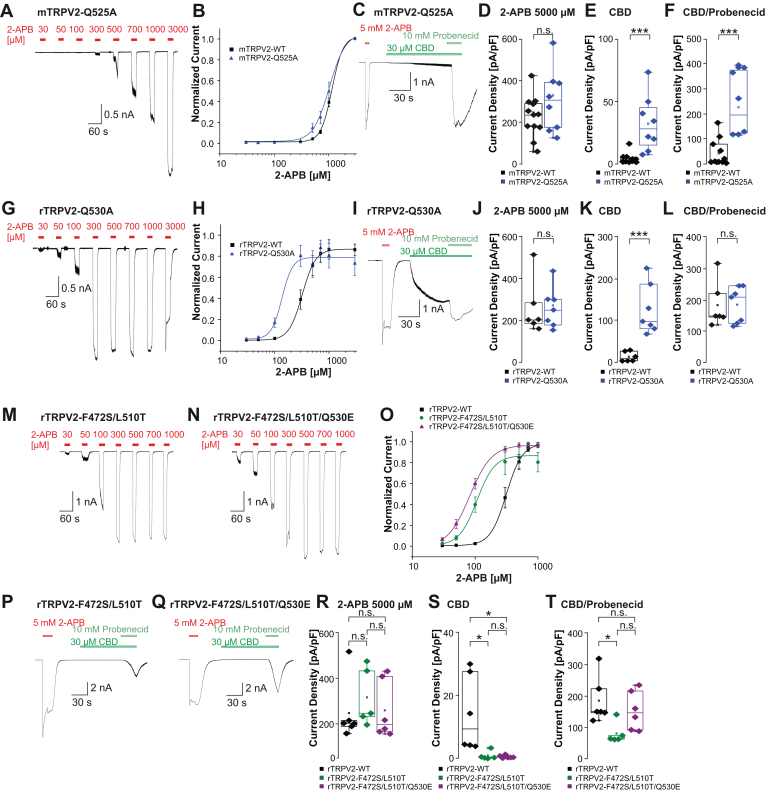
Modification of cholesterol binding residues increases sensitivity to 2-APB, CBD, and probenecid. (A and G) Representative patch clamp traces showing concentration-dependent activation of mTRPV2-Q525A (A) and rTRPV2-Q530A (G) by 2-APB. Increasing concentrations of 2-APB were applied and cells were held at –60 mV. (B and H) Concentration-response curves for mTRPV2 (B) and rTRPV2 (H) for activation by 2-APB. Current amplitudes were measured at each concentration and normalized to the maximum amplitude. Data are presented as mean ± SEM, and the drawn lines represent fits with the Hill equation. (C and I) Samples of patch clamp recordings displaying activation by 5 mM 2-APB followed by 30 *μ*M CBD and the coapplication with 10 mM probenecid in mTRPV2-Q525A (C) and rTRPV2-Q530A (I). (D–F) Comparisons of current densities generated by mTRPV2 WT and -Q525A evoked by 5000 *μ*M 2-APB (D), 30 *μ*M CBD (E), or 30 *μ*M CBD + 10 mM probenecid (F). (J–L) Comparisons of current densities generated by rTRPV2 WT and -Q530A evoked by 5000 *μ*M 2-APB (J), 30 *μ*M CBD (K), or 30 *μ*M CBD + 10 mM probenecid (L). (M and N) Patch clamp traces showing concentration-dependent activation of rTRPV2-F472S/L510T (A) and rTRPV2-F472s/L510T/Q530E (B) by 2-APB. Increasing concentrations of 2-APB were applied and cells were held at –60 mV. (O) Concentration-response curves for experiments displayed in panels A through B. Current amplitudes were measured at each concentration and normalized to the maximum amplitude. (P and Q) Samples of patch clamp recordings displaying activation by 5 mM 2-APB followed by 30 *μ*M CBD and the coapplication with 10 mM probenecid in rTRPV2-F472S/L510T(P) and rTRPV2-F472S/L510T/Q530E (Q). (R–T) Comparisons of current densities evoked by 5000 *μ*M 2-APB (R), 30 *μ*M CBD (S), or 30 *μ*M CBD + 10 mM probenecid (T). Boxes represent the median (50th percentile) along with the 25th and 75th percentiles, whereas the whiskers indicate the 5th and 95th percentiles. Data points outside of the whiskers are plotted individually. ∗*P* < .05; ∗∗*P* < .01; ∗∗∗*P* < .001; and n.s. not significant. *P*-values from the post hoc Tukey test are shown on the plots.

The phenotypes of the investigated mutants of mTRPV2 were not perfectly matched by the phenotypes of the corresponding mutants of rTRPV2, but they all exhibited a loss of function when applied to all 3 drugs tested. In order to examine if a gain-of-function mutation within the VBP also applies to all 3 drugs, we tested mTRPV2-Q525A which was demonstrated to display increased responses to 2-APB.^27^ We observed a small leftward shift of the 2-APB concentration-response curve for mTRPV2-Q525A (*n* = 8) as compared with mTRPV2-WT (*n* = 9, Fig. 5, A and B). The equivalent Gln530Ala mutant in rTRPV2 (EC_50_ = 136 ± 2 *μ*M, *n* = 8) exhibited a clear leftward shift of the concentration-response curve to 2-APB as compared with rTRPV2-WT (Fig. 5, G and H). The current density at 5000 *μ*M 2-APB did not show a significant difference between mTRPV2-WT and mTRPV2-Q525A (Fig. 5, C and D, *n* = 8–12, Student’s *t* test, *P* = .19, 95% CI: –41.44 to 193.2) or rTRPV2-WT and rTRPV2-Q530A (Fig. 4, I and J, *n* = 6–7, Student’s *t* test, *P* = .83, 95% CI: –126.4 to –154.3). However, mTRPV2-Q525A exhibited a significant increase in CBD-induced currents (Fig. 5E, *n* = 8–11, Student’s *t* test, *P* = .0007, 95% CI: 13.98 to 42.77), as well as in currents induced by the combination of CBD and probenecid (Fig. 5F, *n* = 8–11, Student’s *t* test, *P* = .0001, 95% CI: 136.8 to 321). Consistently, experiments with rTRPV2-Q530A showed a significant increase in CBD-induced currents (Fig. 5K, *n* = 6–7, Student’s *t* test, *P* = .001, 95% CI: 56.17 to 165.4). Currents induced by the combination of CBD and probenecid did not differ significantly between rTRPV2-WT and Q530A, probably because rTRPV2-Q530A was already fully activated by CBD alone (Fig. 5L, *n* = 6–7, *P* = .97, 95% CI: –78.62 to 81.9). These data are consistent with the idea that any perturbations within the VBP may have a general effect on the global channel function for both mTRPV2 and rTRPV2. To further assess this hypothesis, we examined the rTRPV2 mutants rTRPV2-F472S/L510T and rTRPV2-F472S/L510T/Q530E. In these mutants, residues within the VBP were replaced with the corresponding residues in the capsaicin receptor TRPV1 (Supplemental Fig. 2). As a result, both mutants were activated by the ultra-potent vanilloid resiniferatoxin.^35^^,^^36^ Again, cells held at –60 mV were treated with increasing concentrations of 2-APB (30–1000 *μ*M) to generate concentration-response curves. Surprisingly, both mutants displayed a pronounced leftward shift in 2-APB sensitivity compared with rTRPV2-WT. rTRPV2-F472S/L510T exhibited an EC_50_ of 106.9 ± 9.8 *μ*M (*n* = 5, Fig. 5, M and O), whereas rTRPV2-F472S/L510T/Q530E showed an even higher 2-APB sensitivity (EC_50_ 81.8 ± 0.8 *μ*M, *n* = 5, Fig. 5, N and O). At a saturating concentration of 2-APB (5000 *μ*M), the current densities did not differ significantly between rTRPV2-F472S/L510T, rTRPV2-F472S/L510T/Q530E, and rTRPV2-WT (Fig. 5, P–R, *n* = 5–6, one-way ANOVA, F(2,14) = 0.44, Tukey HSD post hoc test, WT vs F472S/L510T 95% CI: –273.7 to 135.4; WT vs F472S/L510T/Q530E 95% CI: –207.1 to 183; F472S/L510T vs F472S/L510T/Q530E 95% CI: –147.5 to 261.6). In contrast, both mutants showed strongly diminished CBD sensitivities (Fig. 5, P, Q, and S, *n* = 5–6, one-way ANOVA F(2,14) = 6.6, Tukey HSD post hoc test, WT vs F472S/L510T 95% CI: 1.709 to 24.7; WT vs F472S/L510T/Q530E 95% CI: 2.568 to 24.49; F472S/L510T vs F472S/L510T/Q530E 95% CI: -11.17 to 11.82). Although the coapplication of CBD and probenecid elicited strong responses in the rTRPV2-F472S/L510T/Q530E mutant, the rTRPV2-F472S/L510T mutant showed a reduced activation compared with rTRPV2-WT (Fig. 5, P, Q, and T, *n* = 5–6, one-way ANOVA F(2,14) = 4.237, Tukey HSD post hoc test, WT vs F472S/L510T 95% CI: 8.932 to 200.1; WT vs F472S/L510T/Q530E 95% CI: –59.91 to 122.3; F472S/L510T vs F472S/L510T/Q530E 95% CI: –168.9 to 22.27). Considering that published structural data on rTRPV2 have not identified the residues F472S and L510T as relevant for the sensitivities to 2-APB, CBD, or probenecid,^21^^,^^22^^,^^27^^,^^30^^,^^31^ these data support our notion that mutations within the VBP of TRPV2 can result in unpredictable functional phenotypes and thus hamper the meaningfulness of experiments designed to detect or verify specific agonist and antagonist binding sites.

### 3.3 Modification of 2-APB binding residues within the *binding pocket between the S5 helix and the S4-S5 linker*

**Fig. 6:**
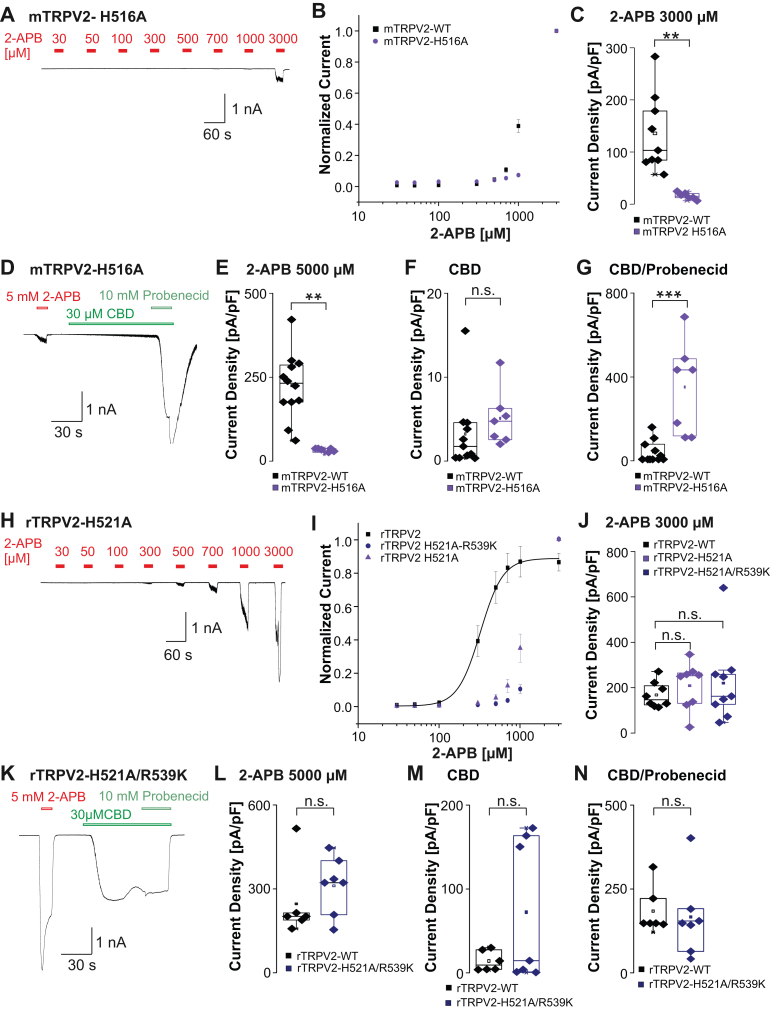
Residues within the S5 binding pocket are relevant for 2-APB sensitivity in both mTRPV2 and rTRPV2. (A) Trace showing concentration-dependent activation of mTRPV2-H516A by 2-APB. Increasing concentrations of 2-APB were applied and cells were held at –60 mV. (B) Concentration-response curves of mTRPV2-WT and mTRPV2-H516A. Current amplitudes were measured at each concentration and normalized to the maximum amplitude. (C) Box plots comparing 2-APB-evoked (3000 *μ*M) current densities in mTRPV2-WT and mTRPV2-H516A. (D) Sample recording displaying activation by 5000 *μ*M 2-APB followed by 30 *μ*M CBD and the coapplication of CBD and 10 mM probenecid on mTRPV2-H516A. (E–G) Comparisons of current densities generated by mTRPV2-WT and mTRPV2-H516A when exposed to 5000 *μ*M 2-APB (E), 30 *μ*M CBD (F), or 30 *μ*M CBD + 10 mM probenecid (G). (H) Trace showing concentration-dependent activation of rTRPV2-H521A by 2-APB. Increasing concentrations of 2-APB were applied and cells were held at –60 mV. (I) Concentration-response curves of rTRPV2-WT, rTRPV2-H516A, and rTRPV2-H516A/R539K. Current amplitudes were measured at each concentration and normalized to the maximum amplitude. (J) Box plots comparing 2-APB-evoked (3000 *μ*M) current densities in rTRPV2-WT, rTRPV2-H516A, and rTRPV2-H516A/R539K. (K) Sample recording displaying activation by 5000 *μ*M 2-APB followed by 30 *μ*M CBD and the coapplication of CBD and 10 mM probenecid on rTRPV2-H516A/R539K. (L–N) Comparisons of current densities generated by rTRPV2-WT and rTRPV2-H516A/R539K when exposed to 5000 *μ*M 2-APB (L), 30 *μ*M CBD (M), or 30 *μ*M CBD + 10 mM probenecid (N). For all displayed box plots, the box represents the median (50th percentile) along with the 25th and 75th percentiles, whereas the whiskers indicate the 5th and 95th percentiles. Data points outside of the whiskers are plotted individually. ∗*P* < .05; ∗∗*P* < .01; ∗∗∗*P* < .001; and n.s. not significant. *P*-values from the post hoc Tukey test are shown on the plots.

We next aimed to investigate the proposed binding of 2-APB in the S5 binding pocket^22^^,^^27^(Supplemental Fig. 2). Patch clamp recordings on mTRPV2-H516A as well as on rTRPV2-H521A and rTRPV2-H521A/R539K were performed.^22^^,^^27^ 2-APB-induced activation of mTRPV2-H516A was strongly reduced with only small inward currents at 3000 *μ*M 2-APB (Fig. 6, A–C, *n* = 6–9, Student s *t* test, *P* = .0018, 95% CI: –186.3 to –53.74). When exposed to a single application of 5000 *μ*M 2-APB, mTRPV2-H516A only produced small inward currents <600 pA (Fig. 6, D and E, *n* = 7–12, Student’s *t* test, *P* = .0012, 95% CI: –184.7 to –54.99). mTRPV2-H516A produced large inward currents when exposed to 30 *μ*M CBD, but this apparent increase of CBD-induced currents was not statistically significant when compared with rTRPV2-WT (Fig. 6, D and F, Student’s *t* test, *P* = .3546, 95% CI: –2.275 to 5.993). However, the combined application of 30 *μ*M CBD and 10 mM probenecid induced a significant gain-of-function phenotype (Fig. 6, D, and G, Student’s *t* test, *P* = .0003, 95% CI: 165.9 to 451.9). Thus, replacement of His516 in mTRPV2 almost fully diminished the sensitivity to 2-APB, while even increasing current induced by CBD and probenecid. As previously described, the 2-APB concentration-response curves for rTRPV2-H521A (*n* = 8) and rTRPV2-H521A/R539K (*n* = 9) were strongly shifted when compared with rTRPV2-WT (Fig. 6, H and I; Supplemental Fig. 3C).^22^ However, both mutants generated robust 2-APB-induced currents that were not significantly smaller than currents observed for rTRPV2-WT (Fig. 6J, *n* = 8–9, one-way ANOVA, F(2,22) = 0.3, Tukey HSD post hoc test, WT vs H521A 95% CI: –200 to 120.7; WT vs H521A/R539K 95% CI: –208.9 to 102.8). It should be noted that current density is not only influenced by channel gating but also by the number of channels expressed at the cell surface, which may be affected by specific mutations. Although rTRPV2-H521A exhibits reduced sensitivity to 2-APB, its current density at 3 mM 2-APB is comparable to rTRPV2-WT. This could reflect a preserved efficacy of 2-APB, but possibly also an altered membrane expression. Although our data strongly suggest a role for His521 in dictating the 2-APB sensitivity of rTRPV2, we did not determine if this mutation also influences membrane expression. This notion also applies for the application of 5000 *μ*M 2-APB, eg, rTRPV2-WT and rTRPV2-H521A/R539K produced large inward currents that were not significantly different (Fig. 6, K and L, *n* = 6–7, Student’s *t* test, *P* = .35, 95% CI: –79.3 to 208.2). Furthermore, rTRPV2-H521A/R539K generated large inward currents when exposed to 30 *μ*M CBD alone or in combination with probenecid (Fig. 6, K, M, and N, Student’s *t* test, *P* = .76, 95% CI: 18.62 to 135.2). These data show that the mutations within the S5 binding pocket of both mTRPV2 and rTRPV2 result in a loss of function that is rather selective for 2-APB sensitivity. The sensitivities to CBD and probenecid were intact or even slightly increased, thus possibly indicating that these sites are relevant for the global function of TRPV2 as well.

### 3.4 2-APB insensitivity of rTRPV2 following replacement of both binding sites

**Fig. 7:**
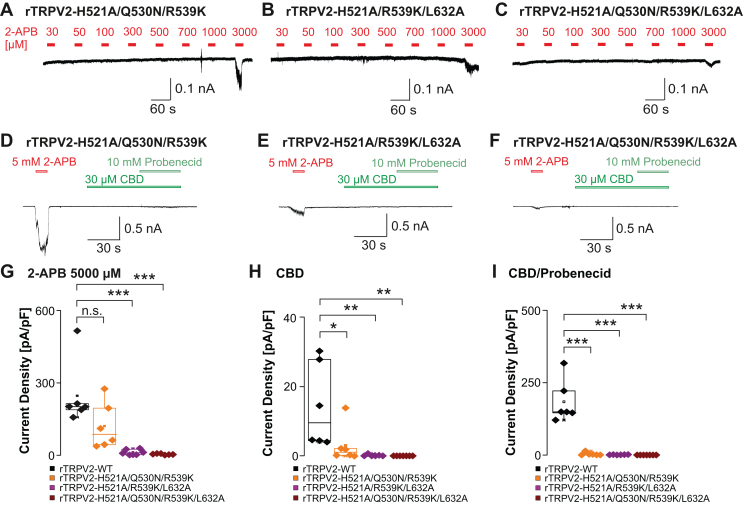
Complete 2-APB insensitivity of rTRPV2 following replacement of both binding sites. (A–C) Patch clamp traces showing concentration-dependent activation of rTRPV2-H521/Q530N/R539K (A), rTRPV2-H521A/R539K/L632A (B), and rTRPV2-H521A/Q530N/R539K/L632A (C) by 2-APB. Increasing concentrations of 2-APB were applied and cells were held at –60 mV. (D–F) Samples of patch clamp recordings displaying activation by 5 mM 2-APB followed by 30 *μ*M CBD and the coapplication with 10 mM probenecid in rTRPV2-H521/Q530N/R539K (D), rTRPV2-H521A/R539K/L632A (E), and rTRPV2-H521A/Q530N/R539K/L632A (F). (G–I) Comparisons of current densities evoked by 5000 *μ*M 2-APB (G), 30 *μ*M CBD (H), or 30 *μ*M CBD + 10 mM probenecid (I). Boxes represent the median (50th percentile) along with the 25th and 75th percentiles, whereas the whiskers indicate the 5th and 95th percentiles. Data points outside of the whiskers are plotted individually. ∗*P* < .05; ∗∗*P* < .01; ∗∗∗*P* < .001; and n.s. not significant. *P*-values from the post hoc Tukey test are shown on the plots.

Our data indicate that rTRPV2 but not mTRPV2 retained substantial 2-APB sensitivity in all mutant constructs on putative 2-APB binding sites (Supplemental Fig. 4). Therefore, we finally generated rTRPV2 mutants lacking key amino acids from both regions suggested to bind 2-APB. Since our data showed that the rTRPV2-Q530N and rTRPV2-L632A mutants were less sensitive to 2-APB than rTRPV2-Y471A, we replaced Gln530 and/or Leu632 in the mutant rTRPV2-H521A/R539K to obtain rTRPV2-H521A/Q530N/R539K, rTRPV2-H521A/R539K/L632A, and rTRPV2 H521A/Q530N/R539K/L632A. The mutant rTRPV2-H521A/Q530N/R539K exhibited small inward currents only when exposed to 3000 and 5000 *μ*M 2-APB (Fig. 7, A and D, *n* = 6–10). The mutants rTRPV2 H521A/Q530N/R539K/L632A and rTRPV2-H521A/Q530N/R539K/L632A displayed almost no 2-APB-induced currents up to 5000 *μ*M (Fig. 7, B, C, E, and G, *n* = 6–7, one-way ANOVA, F(3,21) = 12.34, Tukey HSD post hoc test. WT vs H521A/Q530N/R539K 95% CI: –3.753 to 254.8; WT vs H521A//R539K/L623A 95% CI: 109.6 to 358.8; WT vs H521A/Q530N/R539K/L623A 95% CI: 113.1 to 371.7). As expected, all 3 mutants showed almost no sensitivity to 30 *μ*M CBD and 10 mM probenecid (Fig. 7, D–F, H, and I, one-way ANOVA, F(3,21) = 39, Tukey HSD post hoc test, WT vs H521A/Q530N/R539K 95% CI: 0.531 to 21.26; WT vs H521A//R539K/L623A 95% CI: 4.195 to 24.17 ; WT vs H521A/Q530N/R539K/L623A 95% CI: 3.676 to 24.41). As the residual small currents induced by 5000 *μ*M 2-APB is indicative for a residual channel functionality, these data might suggest that 2-APB activates TRPV2 by interacting with both binding sites.

## 4 Discussion

In this study, we re-examined the role of several residues found in 2 binding pockets that have been suggested to be critical for TRPV2 sensitivity to 2-APB. Although the investigated residues are conserved in rTRPV2, mTRPV2, and even hTRPV2, 2 recent reports have presented data suggesting distinct and possibly species-specific mechanisms for 2-APB sensitivity. TRPV2 has been implicated in numerous pathophysiological processes, including heart failure and various cancers, with early studies demonstrating that TRPV2 modulators binding to the S4/S5 linker or the VBP can suppress glioblastoma progression or reduce prostate cancer metastasis.^12^^,^^16^^,^^18^ With the perspective that structure-based drug screening has the potential to guide the development of therapeutically useful modulators of TRPV2, differences between species commonly used for preclinical research need to be carefully investigated.

Our findings suggest that the mechanisms that encode for 2-APB sensitivity in mTRPV2 and rTRPV2 are conserved, with no detectable species-specific differences at the predicted binding sites. However, a key difference between rTRPV2 and mTRPV2 is the 3-fold higher sensitivity of rTRPV2 (EC_50_ = 322 *μ*M) compared with mTRPV2 (EC50 >1000 *μ*M), consistent with previous reports.^16^^,^^22^^,^^27^ Some previous studies reported even higher 2-APB sensitivities (EC_50_ ∼20 *μ*M) for both orthologs, and this may be due to the use of 2.5 mM probenecid to load cells with the calcium dye.^25^^,^^37^ Additionally, both rodent channels have a greater 2-APB response than hTRPV2, which is only weakly activated by 2-APB at even the highest concentrations. This poor functionality for hTRPV2 is well known, and some studies have even described hTRPV2 as insensitive to 2-APB.^25^^,^^38^ The structural basis for this phenotype of hTRPV2 remains unknown, but it has important implications for interpreting TRPV2 studies across species. Although the key binding pockets examined in this study are conserved in all 3 orthologs, TRPV2 from different species might be differentially regulated by yet unknown cellular. For example, our findings suggest that cholesterol depletion reduces 2-APB-induced currents generated by hTRPV2. Thus, cholesterol may play a facilitating role in hTRPV2 function rather than acting as an inhibitor as described for mTRPV2, TRPV1, and TRPV4.^39^, ^40^, ^41^ This observation would point to a unique regulatory mechanism in hTRPV2, where cholesterol potentially stabilizes the open channel state or facilitates ligand binding. This note remains highly speculative and requires further investigation.

We argue that our data are sufficient to support the assumption that 2-APB seems to bind to the S5 binding pocket to activate both rTRPV2 and mTRPV2. This is supported by our data showing that mutations of the S5 binding pocket reduce 2-APB sensitivity without reducing responses to CBD and probenecid. Although these results align with our originally proposed 2-APB binding site, subsequent reports from other groups have failed to confirm this site in both mTRPV2 and rTRPV2.^27^^,^^31^ The reasons for these discrepancies remain unclear, but our structural data consistently show additional density at the pocket between S5 and the S4-5 linker upon 2-APB binding, along with a conformational change creating this pocket.^22^^,^^29^^,^^30^

Our functional data neither confirm nor refute the proposed binding of 2-APB within the VBP. However, some observations allow us to be critical of this mechanism. First, our data clearly show that the general function of TRPV2 depends on a mechanism at the VBP, possibly related to lipid dynamics. All investigated VBP mutations predicted to have an impact on 2-APB sensitivity displayed reduced sensitivities to all 3 agonists or, in the case of the Q530A/Q525A mutants, increased sensitivities to all 3 agonists. Thus, the mutated residues have a strong global role for TRPV2 function, and not specifically for 2-APB sensitivity. Still, these data do not speak against the binding of 2-APB within the VBD. Second, Su et al^27^ suggested that 2-APB has to displace cholesterol in order to bind to the VBP. We could not reproduce the previously reported increase of 2-APB sensitivity following the depletion of cholesterol, so at least this interaction between cholesterol and 2-APB does not seem to be decisive for 2-APB sensitivity. Third, Su et al^27^ did not observe any conformational changes of mTRPV2 upon 2-APB binding, and their assigned 2-APB densities more closely resemble lipids than the triangular shape expected for 2-APB. As further functional studies will probably not give definitive answers to this uncertainty, further structural studies on rTRPV2 and mTRPV2 seem warranted.

A coincidental observation in this study was the 3–4× fold increase in 2-APB sensitivity of the resiniferatoxin-sensitive rTRPV2 mutants rTRPV2-F472S/L510T and rTRPV2-F472S/L510T/Q530E, where the mutated residues were exchanged by the corresponding residues in TRPV1.^35^^,^^36^ When considering that rTRPV1 displays a much higher sensitivity to 2-APB than TRPV2, these data may suggest that VBP residues that are bound by vanilloids also seem to encode for a high 2-APB sensitivity. If we accept the assumption that 2-APB binds to the VBP of TRPV2, this note seems plausible. However, Gochman et al^31^ demonstrated that the exchange of several other residues within the VBP also results in a change of 2-APB sensitivity. The authors postulated that the region encompassing the VBP is likely to exert allosteric control over the response to 2-APB, an understanding that supports our interpretation of the functional phenotypes of TRPV2 mutants within the VBP.

In conclusion, our data suggest that although there are differences between rTRPV2, mTRPV2, and hTRPV2 sensitivity to several stimuli, they likely share binding sites for 2-APB. Exploring species-specific differences like this is crucial for translational research, as it ensures that findings from model organisms can be accurately applied to human physiology. Although further studies on hTRPV2 are warranted, our findings may aid in the development of selective compounds targeting TRPV2.

## Conflicts of interest

The authors declare no conflicts of interest.
