# Supplementary material for: Molecular determinants of 2-aminoethoxydiphenyl borate sensitivity of transient receptor potential vanilloid 2-unexpected differences between 2 rodent orthologs
Source: Mol Pharmacol. 2025 Jul 11;107(8):100060. doi: 10.1016/j.molpha.2025.100060 (PMC12489355; doi:10.1016/j.molpha.2025.100060)
Supplement: Supplementary Data [file mmc1.pdf]

Molecular determinants of 2-APB-sensitivity of TRPV2 - unexpected differences between two rodent orthologues

Tabea C. Fricke[1,2], Anna Rämisch[1], Ruth A. Pumroy[4], Sebastian Pantke[1,2], Christine Herzog[1], Frank G. Echtermeyer[1], Samer Al-Samir[3,] Volker Endeward[3], Vera Moiseenkova-Bell[4], Andreas Leffler[1]

Affiliations: [1]Department of Anesthesiology and Intensive Care Medicine, Hannover Medical School, 30625 Hannover, Germany. [2]PRACTIS Clinician Scientist Program, Dean's Office for Academic Career Development, Hannover Medical School, Germany. [3]Department of Molecular and Cellphysiology, AG Vegetative Physiologie, Hannover Medical School, 30625 Hannover, Germany. [4]Department of Systems Pharmacology and Translational Therapeutics, Perelman School of Medicine, University of Pennsylvania, Philadelphia, USA

Journal Titel: Molecular Pharmacology

Manuscript number: MOLPHA-D-25-00080R1

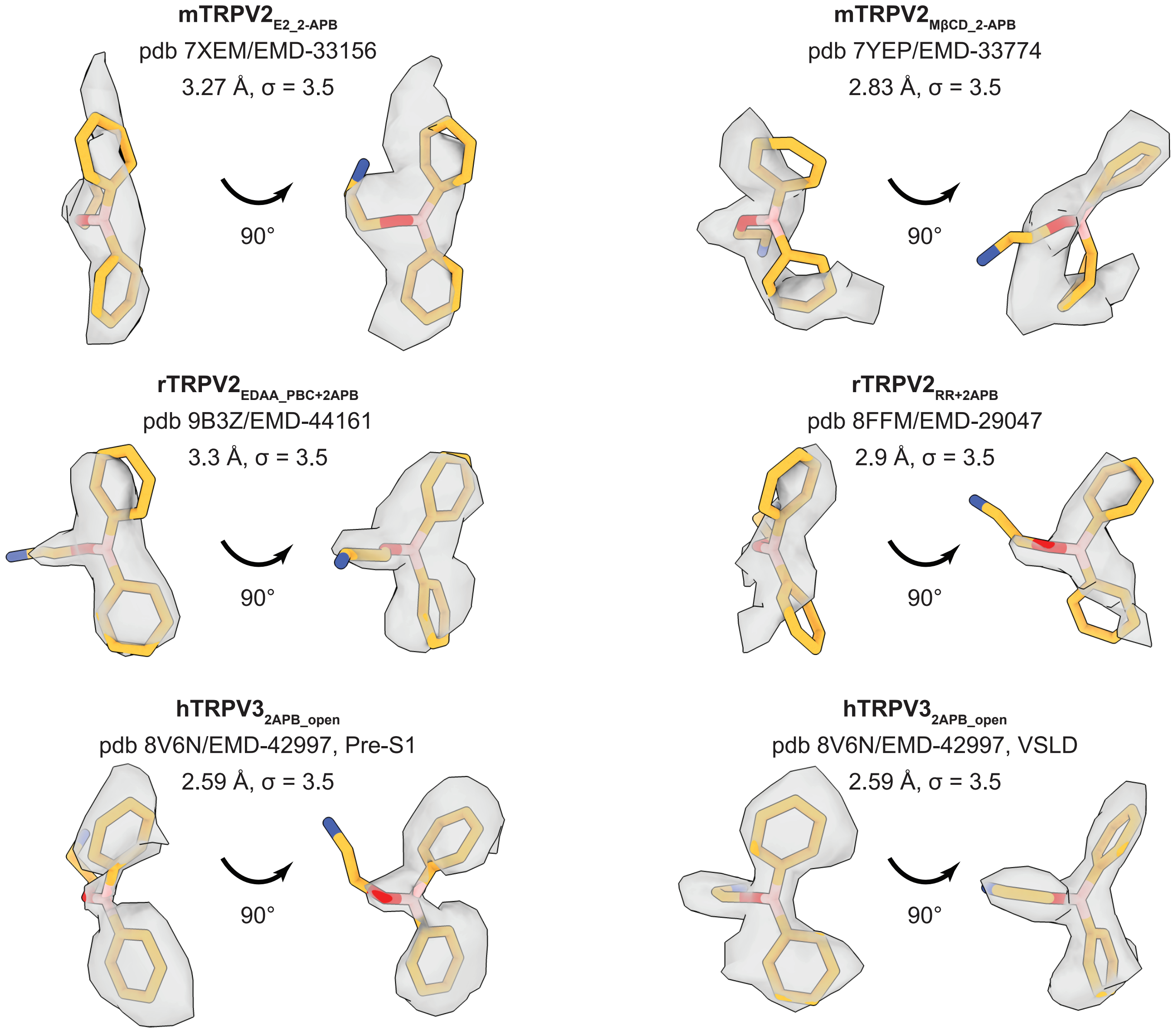

Supplemental Figure 1: Comparison of 2-APB cryoEM density. 2-APB density segmented from full cryoEM maps. Molecules and maps aligned to the central boron atom and the two connected carbons.

```
Rat     TRPV2 460 R-LFIWISFMDSYFEILFLLQALLTVLSQVLRFMETEWYLPLLVLSLVLGWLNLLYYTRGFQHTGIYSVMIQKVILRDLL 538
Mouse   TRPV2 455 R-LFIWISFMDSYFEILFLVQALLTVLSQVLRFVETEWYLPLLVSSLVLGWLNLLYYTRGFQHTGIYSVMIQKVILRDLL 533
Human   TRPV2 458 H-VFIWISFIDSYFEILFLFQALLTVVSQVLCFLAIEWYLPLLVSALVLGWLNLLYYTRGFQHTGIYSVMIQKVILRDLL 536
Human   TRPV1 500 R-PSMKTLFVDSYSEMLFFLQSLFMLATVVLYFSHLKEYVASMVFSLALGWTNMLYYTRGFQQMGIYAVMIEKMILRDLC 578
Human   TRPV3 510 P-SDLQSILSDAWFHFVFFIQAVLVILSVFLYLFAYKEYLACLVLAMALGWANMLYYTRGFQSMGMYSVMIQKVILHDVL 588
Human   TRPV4 536 KcPGVNSLFIDGSFQLLYFIYSVLVIVSAALYLAGIEAYLAVMVFALVLGWMNALYFTRGLKLTGTYSIMIQKILFKDLF 615

Rat     TRPV2 539 RFLLVYLVFLFGFAVALVSLSREARSPKAPED--NNSTVTEQPTVGQEEEP--APYRSILDASLELFKFTIGMGELAFQE 614
Mouse   TRPV2 534 RFLLVYLVFLFGFAVALVSLSREARSPKAPED--SNTTVTEKPTLGQEEEP--VPYGGILDASLELFKFTIGMGELAFQE 609
Human   TRPV2 537 RFLLIYLVFLFGFAVALVSLSQEAWRPEAPTG--PNATESVQPMEGQEDEGNgAQYRGILEASLELFKFTIGMGELAFQE 614
Human   TRPV1 579 RFMFVYIVFLFGFSTAVVTLIEDGKNDSLPSE--STSHRWRGPACRPPDSS----YNSLYSTCLELFKFTIGMGDLEFTE 652
Human   TRPV3 589 KFLFVYIVFLLGFGVALASLIE-----KCPKD--------------NKDCSS---YGSFSDAVLELFKLTIGLGDLNIQQ 646
Human   TRPV4 616 RFLLVYLLFMIGYASALVSLLNPCANMKVCNEdqTNCTVPTYPSCRDSETFS--------TFLLDLFKLTIGMGDLEMLS 687

Rat     TRPV2 615 QLRFRGVVLLLLLAYVLLTYVLLLNMLIALMSETVNHVADNSWSIWKLQKAISVLEMENGYWWCRRKKHREGRLLKVGTR 694
Mouse   TRPV2 610 QLRFRGVVLLLLLAYVLLTYVLLLNMLIALMSETVNSVATDSWSIWKLQKAISVLEMENGYWWCRRKRHRAGRLLKVGTK 689
Human   TRPV2 615 QLHFRGMVLLLLLAYVLLTYILLLNMLIALMSETVNSVATDSWSIWKLQKAISVLEMENGYWWCRKKQ-RAGVMLTVGTK 693
Human   TRPV1 653 NYDFKAVFIILLLAYVILTYILLLNMLIALMGETVNKIAQESKNIWKLQRAITILDTEKSFLKCMRKAFRSGKLLQVGYT 732
Human   TRPV3 647 NSKYPILFLFLLITYVILTFVLLLNMLIALMGETVENVSKESERIWRLQRARTILEFEKMLPEWLRSRFRMGELCKVA-- 724
Human   TRPV4 688 STKYPVVFIILLVTYIILTFVLLLNMLIALMGETVGQVSKESKHIWKLQWATTILDIERSFPVFLRKAFRSGEMVTVGKS 767
```

Supplemental Figure 2: Multiple sequence alignment of TRPV channels. Multiple sequence alignment of TRPV channels highlighting 2-APB interaction sites. Amino acid sequences of rat (rTRPV2), mouse (mTRPV2) and human (hTRPV2) were aligned alongside human TRPV1, TRPV3, and TRPV4 to compare similar and divergent regions relevant to ligand interaction. Residues previously implicated in 2-APB binding within the VBP are highlighted in red, and the residues implicated for RTX-sensitivity in green. Tne residues from the S5 binding domain involved in 2-APB interaction are highlighted in blue. This alignment illustrates the degree of sequence similarity across TRPV family members and highlights the mutation sites investigated in this study.

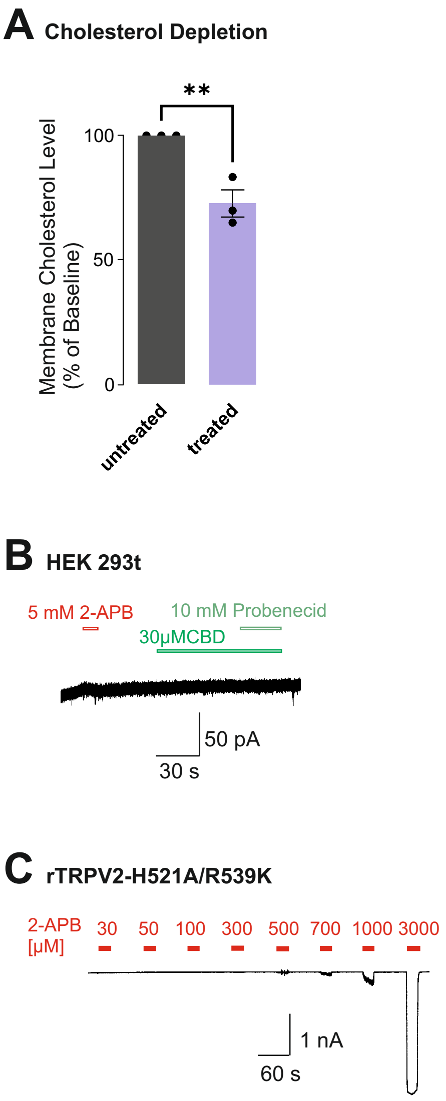

Supplemental Figure 3:

A. Effect of methyl-β-cyclodextrin treatment on membrane cholesterol levels in HEK 293 cells. HEK 293 cells were either left untreated (baseline control, set to 100%) or treated with 10 mM methyl-β-cyclodextrin (MβCD) for 15 minutes to deplete membrane cholesterol. Bars represent mean ± SEM of membrane cholesterol levels expressed as a percentage of the untreated control. The treated group (shown in lila) exhibited a ~27% reduction in membrane cholesterol compared to baseline. **B**. Representative whole-cell patch-clamp current trace displaying an application of 5 mM 2-APB followed by 30 µM CBD and the co-application of 10 mM probenecid in native HEK 293t cells. **C**. Sample current trace from whole-cell patch-clamp experiments showing concentration-dependent activation of the rTRPV2 mutant H521A-R539K by 2-APB. *p< 0.05; **p< 0.01; ***p< 0.001; and n.s. not significant.

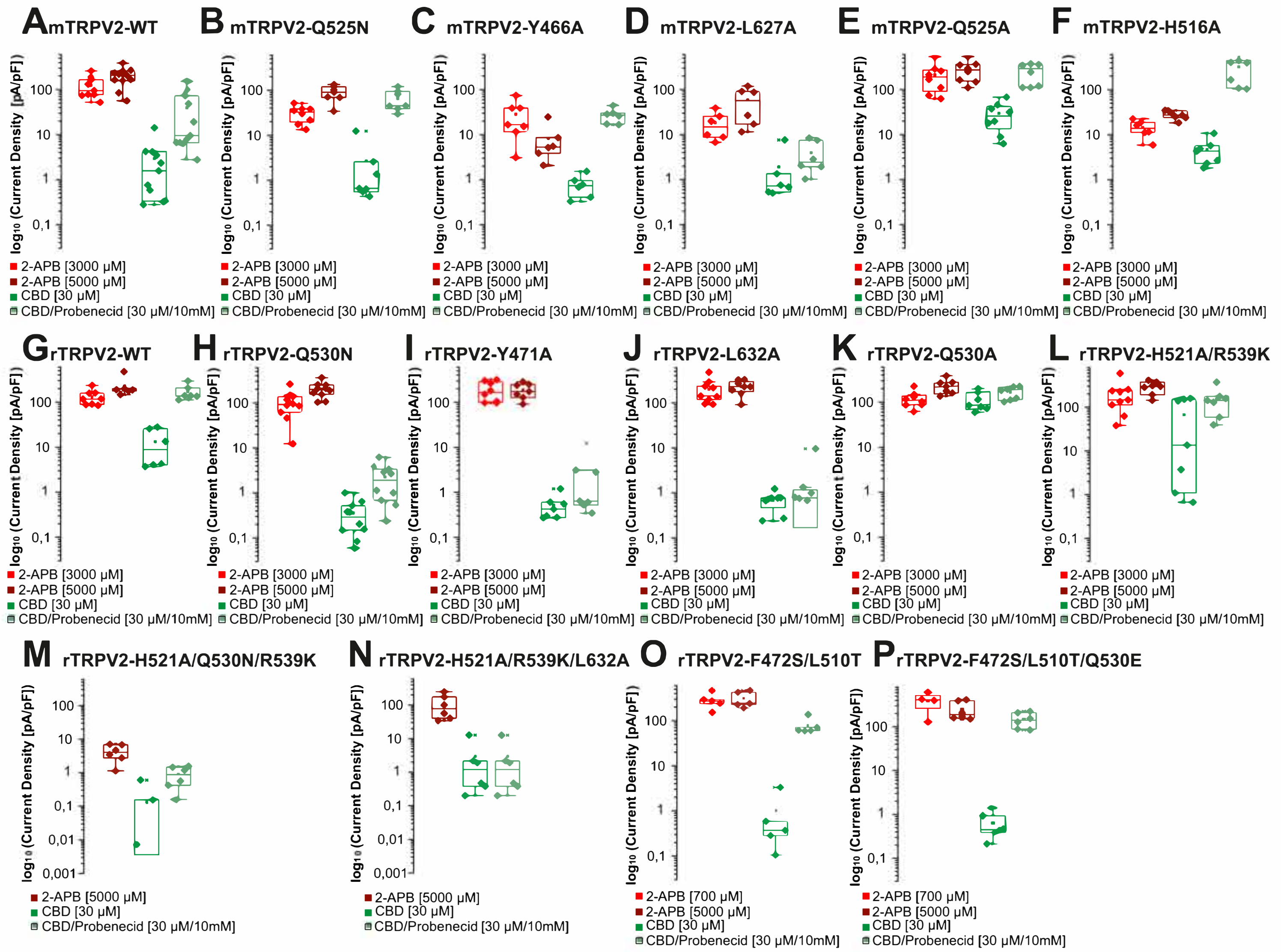

Q

	mouse TRPV2					rat TRPV2						
	VBP				S5 BP	VBP						S5 BP
	-Q525N	-Y466A	-L627A	-Q525A	-H516S	-Q530N	-Y471A	-L632A	-Q530A	-F472S/ L510T	-F472S/ L510T/ Q530E	-H521A/ R539K
2-APB EC_{50}	↓↓↓	↓↓↓	↓↓↓	↑	↓↓↓	↓↓↓	↓	↓↓↓	↑↑↑	↑↑↑	↑↑↑	↓↓↓
2-APB (5mM)	–	↓↓↓	↓	–	↓↓	–	–	–	–	–	–	–
CBD	–	–	–	↑↑↑	–	↓↓↓	↓↓↓	↓↓↓	↑↑↑	↓	↓	–
CBD/Probenecid	–	–	–	↑↑↑	↑↑↑	↓↓↓	↓↓↓	↓↓↓	–	↓	–	–

Supplemental Figure 4: Comparison of current densities evoked by 700 µM or 3000 µM 2-APB (red), 5000 µM 2-APB (brown), 30 µ CBD (dark green), and co-application of CBD (30 µM) with Probenecid (10 mM) (light green) for mouse and rat TRPV2 wild-type and mutant channels. Current densities are plotted on a log-scale to allow for direct comparison across stimuli. Data are shown for mTRPV2 - WT **(A)**, - Q525N **(B)**, - Y466A **(C)**, - L627A **(D)**, - Q525A **(E)**, - H516A **(F)**; rTRPV2 - WT **(G)**, - Q530N **(H)**, - Y471A **(I)**, - L632A **(J)**, - Q530A **(K)**, - H521A/R539K **(L)**, - H521A/Q530N/R539K **(M)**, - H521A/R539K/L632A **(N)**, F472S/L510T **(O)**, - F472S/L510T/Q530E **(P)**.**Q**. The table compares the response of mouse (- Q525N, - Y466A, - L627A, - Q525A, - H516S) and rat (- Q530N, - Y471A, - L632A, - Q530A, - H521A/R539K, - F472S/L510T, - F472S/L510T/Q530E) TRPV2 mutants to wild-type channels under stimulation with 2-APB (estimated EC50 and 5mM), CBD, or the co-application of CBD and probenecid. Arrows indicate the direction and significance of the effect relative to the wild-type of the corresponding species: – = no significant difference; ↓ = p<0.05; ↑↑↑ or ↓↓↓ = p<0.001. Created in BioRender. Leffler, A. (2025) https:// BioRender.com/9pye8mq.
